# GLI1 upregulates C-JUN through a specific 130-kDa isoform

**DOI:** 10.3892/ijo.2013.2222

**Published:** 2013-12-20

**Authors:** LAUREN AMABLE, ELAINE GAVIN, KENJI KUDO, ERHONG MENG, RODNEY P. ROCCONI, LALITA A. SHEVDE, EDDIE REED

**Affiliations:** 1 National Institute on Minority Health and Health Disparities, National Institutes of Health, Bethesda, MD 20892;; 2 Mitchell Cancer Institute, University of South Alabama, Mobile, AL 36604;; 3 Department of Pathology, Comprehensive Cancer Center, University of Alabama at Birmingham, Birmingham, AL 35233, USA

**Keywords:** Hedgehog, GLI1, AP1, C-JUN, cisplatin resistance

## Abstract

The Hedgehog pathway is molecularly linked to increased resistance to cisplatin and increased repair of platinum-DNA damage, through C-JUN. GLI1, which has five known isoforms, is a positive transcriptional regulator in Hedgehog. Southwestern blot assay, EMSA and ChIP assays indicate that only one of five isoforms of GLI1 may be responsible for the Hedgehog link with C-JUN and thus, increased platinum-DNA adduct repair. Cancer tissues express this 130-kDa isoform at levels 6-fold higher than non-malignant tissues; and this isoform exists in abundance in six of seven ovarian cancer cell lines examined.

## Introduction

The importance of the Hedgehog (Hh) signaling pathway in human ovarian cancer is related to its roles in cell invasion and differentiation (1), cellular apoptosis (2) and having an effect on patient prognosis (1,3). The Hh pathway is critically important in the maintenance of human cancer stem cells, in part to the positive transcription factor, GLI1 (4–6). One of the key phenotypic characteristics of cancer stem cells is a high level of drug resistance (1–3,7,8), which may include resistance to platinum compounds (3,9).

Activator protein 1 (AP1), is the positive transcriptional regulator for ERCC1 and other genes of nucleotide excision repair and base excision repair (9,10). Inhibition of AP1 leads to inhibition of nucleotide excision repair (11,12) and sensitization of cells to the anti-cancer agents cisplatin, carboplatin and oxaliplatin (11–14). GLI1 has an important role in regulating C-JUN (15), which participates with C-FOS in the formation of AP1 (16). The role of GLI1 in regulating C-JUN function was first described in keratinocytes by Laner-Plamberger and colleagues (15). They showed that GLI1 and GLI2 directly regulate the expression of C-JUN by binding to a GLI-binding site (GBS) in the C-JUN promoter.

We have investigated the potential role of GLI1, as a determinant of cellular resistance to cisplatin (9). When GLI1 is inhibited by use of an anti-GLI1 shRNA, the following molecular sequence is observed. There is downregulation of mRNA and protein of GLI1 and Sonic Hedgehog, but not GLI2. The C-JUN molecular cascade is switched from a Ser 63/73 cascade to a Thr 91/93 cascade, the latter of which is proapoptotic (17). The normal cisplatin-induced upregulation of DNA repair genes is blocked, resulting in reduced mRNA and protein levels of ERCC1, XPD and XRCC1 (9). Cisplatin-DNA adduct repair is blocked and cells are sensitized to cisplatin by a factor of six (9).

**Figure 1.**
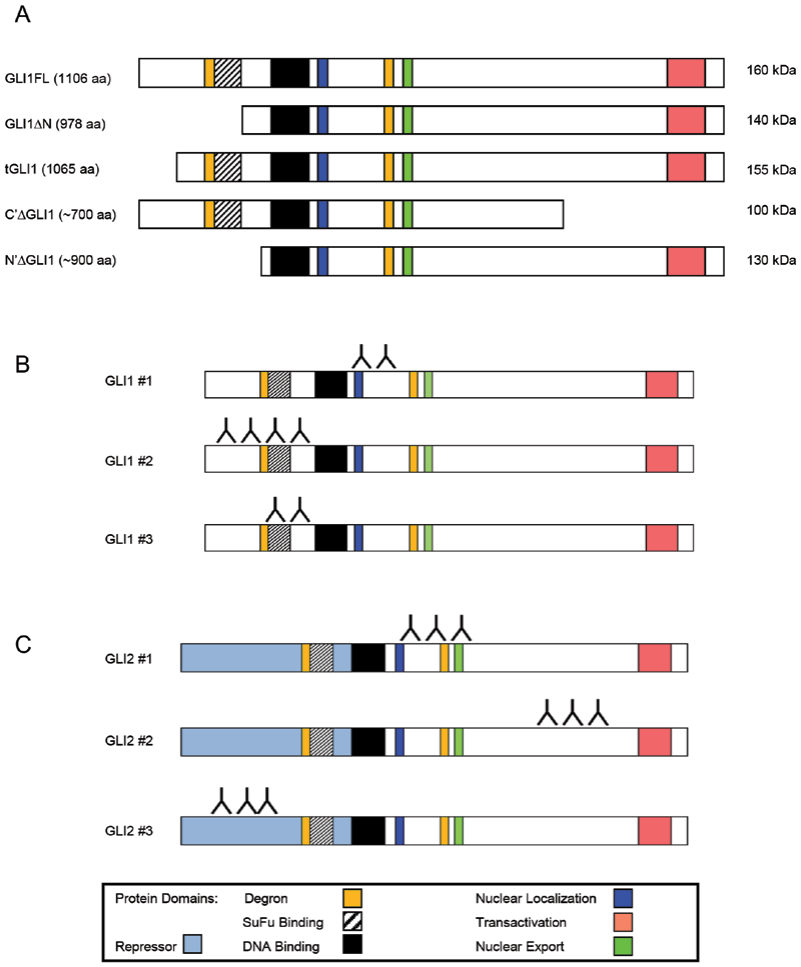
Isoforms and antibodies used in the experiments. (A) The five known isoforms of the GLI1 protein including reported approximate molecular weights. (B) The sites of antibody recognition of the three commercially available anti-GLI1 antibodies used in these studies. (C) The sites of antibody recognition for the three commercially available GLI2 antibodies.

There are five currently known isoforms of the GLI1 protein, shown in Fig. 1A. These isoforms range from ∼700 to 1106 amino acids in length; and, range in molecular weight from 100 to 160 kDa. Two are splice variants of the full-length mRNA. At least one isoform is a post-translational N-terminal truncation of the full-length protein (18). We sought to investigate which isoform of GLI1 is responsible for regulating C-JUN, a known regulator of several genes within nucleotide excision repair and base excision repair. We present in this report our findings, which suggest that only one of the five known isoforms of GLI1 binds the GBS in the promoter of C-JUN.

## Materials and methods

### Cell culture

Cisplatin-sensitive A2780 human ovarian cancer cells and cisplatin-resistant A2780-CP70 and A2780-CIS human ovarian cancer cells were retrieved from frozen stock and used between passages 5 and 30. RPMI-1640 media (Gibco/Invitrogen, Carlsbad, CA, USA) was used to culture cells with the following additives: 10% fetal bovine serum (Gibco), l-glutamine (Gibco), insulin (Sigma-Aldrich, St. Louis, MO, USA) and penicillin/streptomycin (Gibco).

### Electrophoretic mobility shift assay (EMSAs)

Nuclear lysates for electrophoretic mobility shift assays (EMSAs) were prepared from A2780-CP70 cells and the protein concentration determined as described previously (9). Oligonucleotides containing the C-JUN promoter GLI-binding-site (GBS) were purchased with and without a 5′-biotin label: forward, 5′-CTCA ACGTGGGGGGCCGACTCTCG-3′ and reverse, 5′-CGAGAGTCGGCCCCCCACGTTGAG-3′. Double-stranded DNA (dsDNA) probes were generated by adding 1 *μ*M of the forward and reverse compliment oligonucleotides in annealing buffer (10 mM Tris, 1 mM EDTA, 50 mM NaCl, pH 8.0), heated to 95°C and then cooled at a rate of 1°C/min to room temperature.

The DNA-binding reaction was carried out using 20 fmol of biotin-labeled dsDNA, 1 *μ*g poly(dI-dC) and 20 *μ*g nuclear lysate protein in a 20 *μ*l volume of reaction buffer (40 mM HEPES, 25 mM KCl, 10 mM MgCl_2_, 10 mM ZnSO_4_, 500 *μ*M EDTA, 10% glycerol, pH 7.8) on ice for 30 min. In competition experiments, excess unlabeled C-JUN GBS or unlabeled consensus (forward, 5′-CTCAACGGACCACCCAGACTAT CG-3′; reverse, 5′-CGATAGTCTGGGTGGTCCGTTGAG-3′) dsDNA were incubated concurrently at levels 50-, 100- and 200-fold in excess in the binding reaction. In steric hindrance experiments, GLI1 antibodies (using GLI1 nos. 2 and 3 listed below in western blotting) and nuclear lysate were incubated on ice for 30 min prior to the DNA-binding reaction. DNA-protein complexes were separated on a native polyacryl-amide gel, transferred to a positively charged PVDF membrane (BrightStar Plus, Ambion, Austin, TX, USA) and probe binding visualized using the Light Shift Chemiluminescent EMSA kit (Thermo Pierce, Rockford, IL, USA).

### Western and southwestern blotting

Nuclear and whole cell lysates were isolated as previously described (9). The laboratory of Dr Rodney Rocconi kindly provided protein lysates from the human ovarian cancer cell lines: SKOV3, OV-90, ES-2 and TOV-112D. A random sample of seven human ovarian cancer and three non-cancer ovary samples were obtained from the Mitchell Cancer Institute Bio-Bank. Protein was isolated using TRIzol (Invitrogen) according to the manufacturer’s instructions. Approximately 10 *μ*g of patient samples protein lysate was loaded per lane on each gel.

The following primary antibodies were used: α-tubulin (Santa Cruz Biotechnology, Santa Cruz, CA, USA), GLI1 [Cell Signaling (Danvers, MA, USA) 2553; R&D (Minneapolis, MN, USA) AF3455; BioLegend (San Diego, CA, USA) 642401] or GLI2 [Santa Cruz Biotechnology sc-20291; Santa Cruz Biotechnology sc-28674; Abcam (Cambridge, MA, USA) ab26506]. The next day, protein bands were visualized by chemiluminescence (Thermo-Pierce Super Signal West Dura Extended Duration Substrate) using the following HRP-secondary antibody: anti-goat (Promega, Madison, WI, USA), anti-rabbit, or anti-mouse (Cell Signaling). Membrane images were recorded using a Fuji LAS-3000 Intelligent Darkbox Digital Imager.

Protein bands were quantitated by densitometry using the Fuji Image Gauge Software and GLI1 values of each sample were normalized to α-tubulin. For patient samples, the values were normalized to an A2780-CP70 protein standard and expressed as relative ratio to account in differences in each western blotting. The results were then analyzed by two-sided t-test using GraphPad Prism software (GraphPad, La Jolla, CA, USA).

For southwestern blotting, a combination of western and Southern blotting techniques was employed to characterize the protein-DNA interactions of the C-JUN GBS based on the method of Cheng *et al* (19). Approximately 60 *μ*g of nuclear lysate was loaded per lane and transferred to a PVDF membrane. Each lane was individually cut from the membrane and subjected either to western blotting with GLI1 or GLI2 antibodies, or southwestern blotting with the C-JUN dsDNA biotin-labeled probe. Proteins were renatured by incubating the membrane in 5% non-fat milk/TNED buffer (10 mM Tris, 50 mM NaCl, 0.1 mM EDTA, 1 mM DTT, pH 7.8) and incubated overnight with 5 nM C-JUN GBS in 5% milk/TNED at 4°C. The protein-DNA bands were visualized using Streptavidin-HRP Conjugates using the Light Shift Chemiluminescent EMSA kit.

### Plasmids, transfections and immunoprecipitation

Full-length GLI1 cDNA was obtained in plasmid form as a gift of Bert Vogelstein (Addgene plasmid no. 16419, Cambridge, MA, USA). GLI1 was MYC-tagged (EQKLISEEDL) on the C-terminal end of the protein by PCR using the primers: forward, 5′-AAAAAAAAAAGCTTATGTTCAACTCGAT GACCCCA-3′; reverse, 5′-AAAAAAAAAAAGCTTCTACAGATCTTCTTCAGAAATAAGTTTTTGTTCGGCACTAGAG TTGAGGAATTC-3′. The resulting fragment was digested with *Hin*dIII and cloned into the pLNCX vector (Clonetech, Mountainview, CA, USA). Verification of the insert and orientation was done by sequencing.

GLI1-MYC was transfected into A2780-CP70 cells using FuGENE6 (Roche, Indianapolis, IN, USA). At 24 h post-transfection, GLI1-MYC transfected cells were lysed in 1% Triton X-100, 50 mM Tris-HCl pH 7.2, 150 mM NaCl, protease inhibitor cocktail (Sigma) and PhosSTOP (Roche). Approximately, 1,000 *μ*g GLI1-MYC protein lysate was immunoprecipitated overnight using Protein A/G Plus-Agarose beads (Santa Cruz Biotechnology, sc-2003) and 4 *μ*g MYC antibody (Santa Cruz Technology, 9E10). The next day, the immunoprecipitation was washed and eluted by heating the sample for 5 min at 95°C in 20 *μ*l of 2X Laemmli sample buffer (Bio-Rad, Hercules, CA, USA).

### Chromatin immunoprecipitation assays (ChIPs)

Cells were seeded at 1.8×10^6^ in 10-cm^2^ dishes and transfected with the pLNCX-GLI1-MYC plasmid the following morning. ChIP assays were performed 24 h after transfection using the Thermo Pierce Agarose ChIP kit according to the manufacturer’s instructions. Normal rabbit IgG antibody was used for mock control (provided). Two antibodies were used for GLI1: the Cell Signal GLI1 antibody listed in western methods and a goat polyclonal to GLI1 (C-18, Santa Cruz Biotechnology) which was previously described by Laner-Plamberger *et al* (15). Additionally, the MYC antibody was used to identify the full-length GLI1 and 130-kDa isoform. Real-time PCR was performed on the isolated DNA using Fast Sybr Green Master Mix (Applied Biosystems, Foster City, CA, USA). Primer sequences for amplifying the C-JUN promoter were described previously (15). Fold enrichment was determined by first calculating the non-specific adjustment by subtracting the Ct of the mock from the Ct of each immunoprecipitation (DDCt). Fold enrichment was then determined using the equation 2^−ΔΔCt^. The results shown are from five independent experiments, with each immunoprecipitation ran in triplicate for real-time PCR. Results were analyzed by one-way ANOVA and Tukey’s post test using GraphPad Prism Software.

## Results

### GLI1, not GLI2, is responsible for binding the GBS in the C-JUN promoter

**Figure 2.**
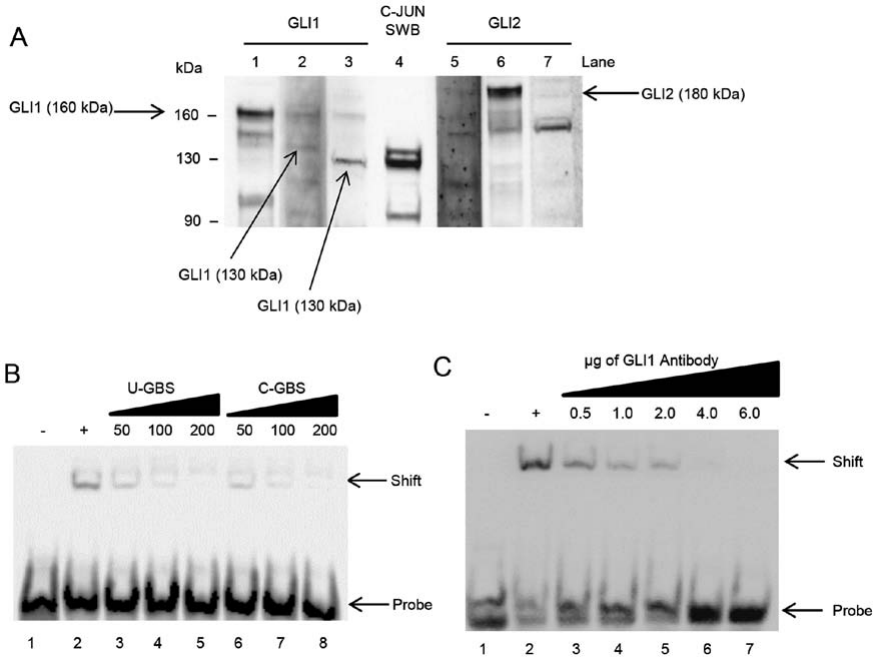
(A) Simultaneous western and southwestern blot analyses show the 130-kDa isoform of GLI1, not GLI2, binds the GBS in the C-JUN promoter in A2780-CP70 cells. Lanes 1–3, are western blot analyses using antibodies to GLI1. Lane 4 is the southwestern blotting of nuclear lysate with the C-JUN promoter GBS. Lanes 5–7, are western blot analyses using GLI2 antibodies. The GLI1 antibodies used in lanes 2 and 3, align with 130 kDa-double band that binds the GBS in the C-JUN promoter. (B) Competition EMSAs using unlabeled C-JUN GBS (U-GBS) and consensus GBS (C-GBS) probes were performed. Adding unlabeled C-JUN GBS or consensus GBS to the reaction at increasing amounts resulted in a stepwise reduction in the band shift signal. (C) Steric hindrance EMSAs using increasing amount of GLI1 antibodies prior to the reaction blocks the C-JUN GBS probe from binding to GLI1, thus reducing the signal.

First, we performed simultaneous western and southwestern blot analyses on A2780-CP70 nuclear lysate, shown in Fig. 2A. Three different GLI1 antibodies (Fig. 1B) were used, shown in lanes 1–3, to detect the different isoforms via western blotting. Additionally, lanes 5–7 are western blot analyses using three GLI2 antibodies (Fig. 1C). Southwestern blotting was performed using biotin labeled dsDNA probe to the GBS found in the C-JUN promoter (lane 4).

The southwestern blotting in lane 4 shows three bands that clearly bind the GBS: a double band at ∼130 kDa and a third band at ∼90 kDa. Anti-GLI1 antibody no. 1 (lane 1); did not recognize a 130-kDa protein in nuclear lysate. The second GLI1 antibody, lane 2, recognizes a band at ∼130 kDa. The western blotting with the third GLI1 antibody, lane 3, recognizes a band at 130-kDa. The two 130-kDa proteins recognized by the second and third GLI1 antibodies, correspond to the 130-kDa double band seen in lane 4. No full-length 160-kDa GLI1 binds the GBS of the C-JUN promoter in A2780-CP70 cells (lane 4).

Western blot analyses using three GLI2 antibodies were performed shown in Fig. 2A, lanes 5–7. None of the three GLI2 antibodies recognized a 130- or 90-kDa molecular weight protein such as those shown in lane 4. Although GLI2 can be a positive transcriptional regulator for C-JUN (15), there is no protein band in lanes 5–7, that correspond to the positive double band at 130-kDa in lane 4.

Next, we performed experiments to confirm a GLI protein in A2780-CP70 cells specifically binds the GBS in the C-JUN promoter. We performed two different EMSA approaches to address this question. The first EMSA approach was to perform DNA-binding competition experiments, using unlabeled oligonucleotides to the GBS in the C-JUN promoter, as well as consensus GBS oligonucleotides (15). The second EMSA approach was to sterically inhibit binding of GBS to nuclear lysate protein, using antibodies to GLI1.

In Fig. 2B, we demonstrate successful competition for the GBS in A2780-CP70 cells. We used the GBS from the C-JUN promoter and separately, we used the consensus GBS sequence. In our negative control, lane 1, no nuclear lysate was added with the biotin-labeled C-JUN probe. When nuclear lysate is added we see a band shift, indicating that a GLI protein binds the GBS in the C-JUN promoter. Increasing concentrations of unlabeled C-JUN GBS results in decreased band shift signal, demonstrated in lanes 3–5. As the ratio of unlabeled DNA to labeled DNA increases, signal is reduced in a stepwise fashion. In lanes 6–8, the increase of unlabeled consensus GBS also successfully competed for GLI binding. When unlabeled consensus GBS is increased in a stepwise fashion, the band shift signal is reduced.

In Fig. 2C, we used antibodies to GLI1, to conduct competition experiments to block binding of the DNA probe to nuclear lysate protein. In these experiments, we used a combination of GLI1 antibodies nos. 2 and 3 which corresponds to the doublet bands seen in the southwestern blotting in Fig. 2A. These antibodies are directed to the N-terminal region of the protein, in the area where the GBS is expected to bind (Fig. 1B). Once bound, the antibodies should occupy the protein and thereby inhibit the binding of GLI1 to the GBS of the C-JUN promoter. In lanes 3–7 the nuclear lysates were pre-incubated with increasing amounts of GLI1 antibodies. As shown in Fig. 2C, when increasing amounts of GLI1 antibody are added, the ability to bind the C-JUN GBS decreases in a stepwise fashion indicating competitive blockage of the DNA-binding domain of GLI1. This suggests GLI1 binds the C-JUN GBS in A2780-CP70 cells.

### The 130-kDa isoform is produced from full-length GLI1 and binds the C-JUN promoter

**Figure 3.**
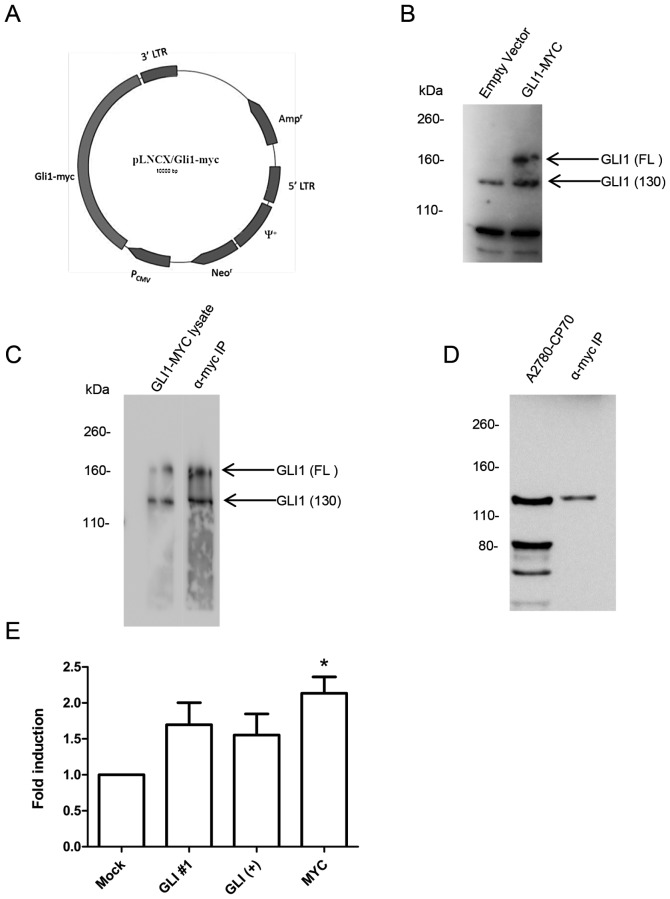
The 130-kDa isoform of GLI1 is produced from the full-length protein and binds the C-JUN promoter. (A) Graphical representation of the C-terminal MYC tag GLI1 construct. (B) Western blotting comparing the empty vector control versus the GLI1-MYC transfected protein lysate. Cells transfected with GLI1-MYC express more full-length GLI1 and the 130-kDa GLI1 isoform. (C) Western blotting of transfected lysate and MYC immunoprecipitation using GLI1 antibody no. 3. Both the full-length and 130-kDa isoform of GLI1 are present in the MYC immunoprecipitation. (D) Southwestern blotting of the MYC immunoprecipitation, showing the 130-kDa GLI1 protein binds the GBS in the C-JUN promoter. (E) ChIP analyses using three antibodies to immunoprecipitate GLI1 to assess binding to the C-JUN promoter using cells transfected with the GLI1-MYC cDNA. GLI1 antibody no. 1 and a positive GLI1 antibody (GLI1^+^) used previously to show binding to the C-JUN promoter (15) recognized both endogenous and transfected GLI1 and isoforms. The MYC antibody recognized only the transfected GLI1-MYC and the 130-kDa GLI1 isoform. The MYC immunoprecipitation had the highest fold induction and was statistically significant (^*^P≤0.05, one-way ANOVA). This suggests the 130-kDa isoform of GLI1 binds the GBS of the C-JUN promoter.

We generated an expression plasmid containing the full-length GLI1 cDNA fused with a C-terminal MYC tag by PCR, shown in Fig. 3A. Transfection of A2780-CP70 cells with the C-terminal MYC tagged GLI1 overexpress full-length GLI1 and the 130-kDa isoform, as compared with empty vector transfected cells, shown in Fig. 3B.

Using protein lysate from transfected cells, immunoprecipitation was performed using the MYC tag antibody. Fig. 3C shows the western blotting comparing whole-cell lysate transfected with GLI1-MYC and the lysate immunoprecipitated with a MYC antibody. The immunoprecipitation of the transfected lysate shows both the full-length and the 130-kDa isoforms of GLI1 were isolated using the MYC antibody. The 130-kDa protein is produced by N-terminal cleavage of the full-length GLI1 (18).

The southwestern blotting was repeated using the MYC immunoprecipitation of transfected cells. Shown in Fig. 3D, no full-length GLI1, corresponding to the 160-kDa protein, was observed to bind the C-JUN GBS. However, a band corresponding to the 130-kDa isoform of GLI1 observed in the nuclear lysate was similarly observed in the MYC immunoprecipitation. This suggests that only the 130-kDa GLI1 isoform binds the GBS of the C-JUN promoter.

The *in vivo* binding of the GLI1 isoform was assessed by performing ChIP assays. Cells were transfected with GLI1-MYC cDNA and three antibodies were used to immunoprecipitate GLI1. The first two antibodies were specific to GLI1 and they recognized both endogenous and transfected GLI1 and GLI1 isoforms. The third antibody was the MYC antibody, which only recognized the transfected GLI1-MYC and the 130-kDa GLI1 isoform but does not recognize any endogenous GLI1 isoforms. The ChIP results are shown in Fig. 3E.

The ChIP using the GLI1 antibody no. 1, which recognized both endogenous and transfected GLI1 and isoforms, had a fold induction of 1.69 over the mock control. The second GLI1 antibody was used as a positive control based on a previous ChIP demonstrating GLI1 binds the C-JUN promoter (15). The ChIP with the GLI1^+^ antibody, which recognized both the endogenous and transfected GLI1 isoforms, had a fold induction of 1.55. The anti-MYC antibody only recognized the transfected GLI1-MYC and the 130-kDa isoform as shown previously in Fig. 3C. The MYC ChIP had the highest fold induction of 2.13 and was statistically significant (P<0.05). Taken together with the results from the southwestern blot analyses, the data strongly suggest that only the 130-kDa isoform recognizes the C-JUN GBS.

### The 130-kDa GLI1 isoform is expressed at higher levels in ovarian cancer

**Figure 4.**
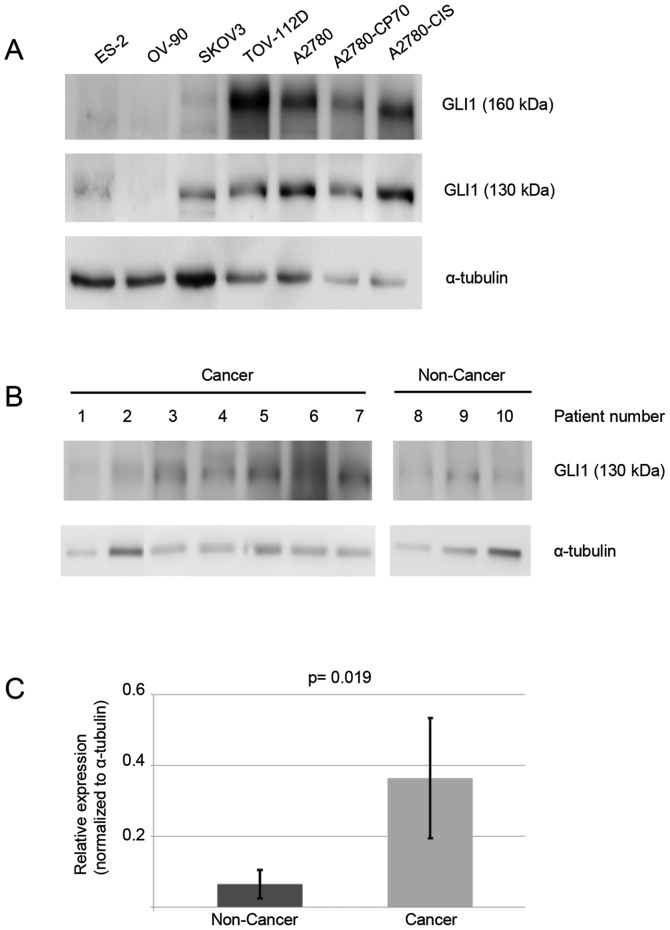
The 130-kDa isoform of GLI1 is present in human ovary cancer specimens. (A) Seven human ovarian cancer cell lines were screened by western blotting for the presence of full-length GLI1 (160 kDa) and the 130-kDa GLI1 isoform. In all cell lines analyzed, the 130-kDa isoform of GLI1 was present. (B) Ten human ovarian specimens, seven cancer and three non-cancer, were assayed by western blotting. In all samples, the 130-kDa isoform of GLI1 was observed. (C) Graphic results of quantification of the 130-kDa protein in patient samples. Protein levels were 6-fold higher in malignant tissues.

In Fig. 4A, six additional human ovarian cancer cell lines were studied for the presence of the 130-kDa GLI1 protein: ES-2, OV-90, SKOV-3, TOV-112D, A2780, A2780-CP70 and A2780-CIS. The cisplatin-resistant cell lines, A2780-CP70 and A2780-CIS, express a higher level of the 130-kDa GLI1 isoform when compared to the parental cisplatin-sensitive A2780 cells. A2780-CP70 cells have a 1.6-fold higher and A2780-CIS a 2.7-fold higher protein level of the 130-kDa GLI1 isoform.

In Fig. 4B, we performed western blot analyses on ten ovary tissue samples from ten separate patients. Seven were cancer specimens, three were non-cancer specimens. The 130-kDa GLI1 isoform was quantitated and non-cancer ovarian samples had an average signal of 0.066. Ovarian cancer samples had an average signal of 0.365, a 6-fold difference (Fig. 4C, P=0.019). Thus, the 130-kDa GLI1 isoform is more highly expressed in ovarian cancer, as opposed to non-cancer ovarian tissues.

## Discussion

The Hh pathway has three groups of transcriptional regulatory proteins: GLI1, GLI2 and GLI3 (4–6,20,21). GLI1 appears to have at least five different isoforms, GLI2 has at least four isoforms and GLI3 has at least five isoforms (20). The transcriptional activity of each of these depends on the specific isoform in question, the specific tissue in question and the tissue context (4–6). Recently, there is a growing interest in the specific functions of the GLI1 isoforms (22,23). GLI1 has many different functions, however, many of these functions are not ascribed to a particular isoform. Stecca and Ruiz i Altaba investigated the 130-kDa isoform of GLI1 in neural stem cells (18). They reported that the 130-kDa isoform is always expressed as a doublet, which are the phosphorylated and unphosphorylated forms of the same protein. In an analysis of a panel of tumor cell lines, the most abundant isoform was the 130-kDa protein and that the relative abundance of three different GLI1 isoforms was: GLI1-130kDa > GLI1-100kDa > GLI1 full length-160-kDa.

The interface between GLI1 and C-JUN has been recognized by several groups (9,15,24). Since GLI1 binds to C-JUN, this suggests that the GLI1 nexus with C-JUN may actually be a GLI1 nexus the AP1 heterodimer and all downstream targets of AP1 and of C-JUN. This strongly suggests that only one of the five known isoforms of GLI1, the 130-kDa isoform, has a role in the modulation of ERCC1 and potentially other genes of nucleotide excision repair (9,10,12,14).

A strong link between GLI1 and the regulation of DNA damage has been reported by Agyeman and colleagues (25), Leonard *et al* (26) and by our group (9). Our study has specifically focused on platinum-based anticancer chemotherapy; and on genes in nucleotide excision repair and base excision repair pathways. The study by Agyeman and colleagues suggest the possibility, that the GLI1 effects on DNA repair response may in fact be more broad than these two specific DNA repair pathways.

The 130-kDa isoform is expressed at higher levels in ovarian cancer specimens than in non-cancer ovarian specimens, suggesting the possibility of the importance of this isoform in the malignancy. We have reported the role of GLI1 as a factor in cellular resistance to cisplatin (9). The data presented here, suggest that the 130-kDa GLI1 isoform is a determinant of cellular resistance to cisplatin. Thus, this specific protein may possibly be a reasonable target for reversing resistance to platinum chemotherapy drugs.

## References

[1] Liao X, Siu MK, Au CW (2009). Aberrant activation of hedgehog signaling pathway in ovarian cancers: effect on prognosis, cell invasion and differentiation. Carcinogenesis.

[2] Chen X, Horiuchi A, Kikuchi N (2007). Hedgehog signal pathway is activated in ovarian carcinomas, correlating with cell proliferation: it’s inhibition leads to growth suppression and apoptosis. Cancer Sci.

[3] Ray A, Meng E, Reed E, Shevde LA, Rocconi RP (2011). Hedgehog signaling pathway regulates the growth of ovarian cancer spheroid forming cells. Int J Oncol.

[4] Mas C, Ruiz i Altaba A (2010). Small molecule modulation of HH-GLI signaling: current leads, trials and tribulations. Biochem Pharmacol.

[5] Stecca B, Ruiz IAA (2010). Context-dependent regulation of the GLI code in cancer by HEDGEHOG and non-HEDGEHOG signals. J Mol Cell Biol.

[6] Zhu H, Lo HW (2010). The human glioma-associated oncogene homolog 1 (GLI1) family of transcription factors in gene regulation and diseases. Curr Genomics.

[7] Mine T, Matsueda S, Gao H (2010). Created Gli-1 duplex short-RNA (i-Gli-RNA) eliminates CD44 Hi progenitors of taxol-resistant ovarian cancer cells. Oncol Rep.

[8] Steg AD, Bevis KS, Katre AA (2012). Stem cell pathways contribute to clinical chemoresistance in ovarian cancer. Clin Cancer Res.

[9] Kudo K, Gavin E, Das S, Amable L, Shevde LA, Reed E (2012). Inhibition of Gli1 results in altered c-Jun activation, inhibition of cisplatin-induced upregulation of ERCC1, XPD and XRCC1 and inhibition of platinum-DNA adduct repair. Oncogene.

[10] Zhong X, Thornton K, Reed E (2000). Computer based analyses of the 5′-flanking regions of selected genes involved in the nucleotide excision repair complex. Int J Oncol.

[11] Bonovich M, Olive M, Reed E, O’Connell B, Vinson C (2002). Adenoviral delivery of A-FOS, an AP-1 dominant negative, selectively inhibits drug resistance in two human cancer cell lines. Cancer Gene Ther.

[12] Li Q, Tsang B, Bostick-Bruton F, Reed E (1999). Modulation of excision repair cross complementation group 1 (ERCC-1) mRNA expression by pharmacological agents in human ovarian carcinoma cells. Biochem Pharmacol.

[13] Reed E (2006). ERCC1 measurements in clinical oncology. N Engl J Med.

[14] Reed E (2010). DNA damage and repair in translational oncology: an overview. Clin Cancer Res.

[15] Laner-Plamberger S, Kaser A, Paulischta M, Hauser-Kronberger C, Eichberger T, Frischauf AM (2009). Cooperation between GLI and JUN enhances transcription of JUN and selected GLI target genes. Oncogene.

[16] Li Q, Gardner K, Zhang L, Tsang B, Bostick-Bruton F, Reed E (1998). Cisplatin induction of ERCC-1 mRNA expression in A2780/CP70 human ovarian cancer cells. J Biol Chem.

[17] Raivich G (2008). c-Jun expression, activation and function in neural cell death, inflammation and repair. J Neurochem.

[18] Stecca B, Ruiz i Altaba A (2009). A GLI1-p53 inhibitory loop controls neural stem cell and tumour cell numbers. EMBO J.

[19] Cheng CK, Yeung CM, Hoo RL, Chow BK, Leung PC (2002). Oct-1 is involved in the transcriptional repression of the gonadotropin-releasing hormone receptor gene. Endocrinology.

[20] Ruiz i Altaba A (1999). Gli proteins encode context-dependent positive and negative functions: implications for development and disease. Development.

[21] Ruiz i Altaba A (2011). Hedgehog signaling and the Gli code in stem cells, cancer and metastases. Sci Signal.

[22] Carpenter RL, Lo HW (2012). Identification, functional characterization and pathobiological significance of GLI1 isoforms in human cancers. Vitam Horm.

[23] Carpenter RL, Lo HW (2012). Hedgehog pathway and GLI1 isoforms in human cancer. Discov Med.

[24] Lo HW, Zhu H, Cao X, Aldrich A, Ali-Osman F (2009). A novel splice variant of GLI1 that promotes glioblastoma cell migration and invasion. Cancer Res.

[25] Agyeman A, Mazumdar T, Houghton JA (2012). Regulation of DNA damage following termination of Hedgehog (HH) survival signaling at the level of the GLI genes in human colon cancer. Oncotarget.

[26] Leonard JM, Ye H, Wetmore C, Karnitz LM (2008). Sonic Hedgehog signaling impairs ionizing radiation-induced checkpoint activation and induces genomic instability. J Cell Biol.

